# Application of Spectral Entropy in Haul Truck Joint Damage Detection

**DOI:** 10.3390/s22197358

**Published:** 2022-09-28

**Authors:** Paweł Stefaniak, Wioletta Koperska, Artur Skoczylas, Maria Stachowiak

**Affiliations:** KGHM Cuprum Research and Development Centre Ltd., Gen. W. Sikorskiego Street 2-8, 53-659 Wroclaw, Poland

**Keywords:** spectral entropy, inertial sensor, damage detection, backlash detection, vibration, truck haul, joint damage

## Abstract

Early detection of machine failures is often beneficial, both financially and in terms of worker safety. The article presents the problem of frequently damaged joints in haul trucks, which are a real threat to the health and life of drivers. It was decided to investigate the problem in terms of dynamic overloads using two NGIMU inertial sensors and placing them in two places on the machine in close proximity to a joint. The data were captured during the standard operation of various machines in several mining departments, which allowed for the detection of a variety of factors influencing vibration. A hypothesis was developed that any changes in the joint would cause a change in the characteristics of vibrations, which were measured using the spectral entropy of vertical vibrations. Analyses have shown that there is a relationship between the change in spectral entropy difference (between the front and back of the vehicle) and joint events: nut tightening, nut replacement, and even joint fracture and replacement. The presented results offer the potential to create a tool for joint diagnostics and the early detection of damage or backlash.

## 1. Introduction

Self-propelled machines are key mining assets used in the horizontal transport of excavated material in underground mines. The process is not complex; the machine transports the output from point A (mining face) to point B (unloading point with screen). In this way, in successive cycles, the material is fed to the further means of transport-belt conveyors, through which it is moved toward the mining shafts and then on the surface directly to the ore enrichment plant. Depending on the distance between points A and B, the process of wheel haulage takes place in various configurations. Bucket loaders are used for short routes (up to around 300 m). In the case of routes exceeding 300 m, the bucket loader loads the haul truck’s cargo box in the mining area, which transports the output to the unloading point with the grid. To ensure continuity of production, the self-propelled machines must meet enormous expectations in terms of performance, reliability and safety. For this reason, special attention has been paid to the monitoring and development of advanced analytics in the area of predictive maintenance. In the literature, we can find many works in the field of efficiency assessment [1,2,3], road conditions assessment [4,5] and technical diagnostics [6,7,8]. Commonly known methods of fault detection mainly concern critical components, such as the engine or gearbox. Most often, these methods are based on temperature and pressure data. It should be highlighted that other types of damage are not considered, particularly in the literature or shown in a marginal manner, one of such being structural damage.

**Figure 1:**
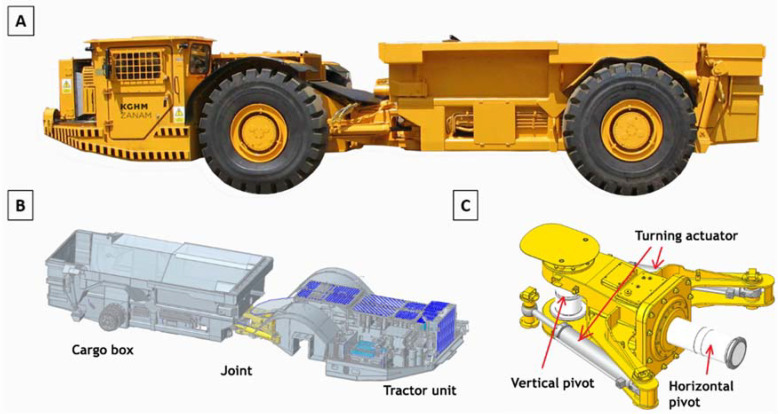
Investigated object: (**A**) haul truck, (**B**) main structural parts, (**C**) the construction of the joint.

Machines working in underground environments are adapted to operations in difficult conditions. One such machine is the haul truck (Figure 1A), which was designed for high capacity along with maneuverability. This is the reason why this particular type of machine consists of two main parts: the driving (tractor) and transport (cargo box). These two parts are connected by the joint, as shown in Figure 1B. The joint of a haul truck (Figure 1C) has two degrees of freedom. The turning mechanism is based on two hydraulic cylinders installed between the two above-mentioned working parts. This design solution allows for crossing intersections at an angle of 90 degrees. The joint is one of the most important structural nodes and its damage is one of the most costly and dangerous. Its reliability mainly depends on the quality of workmanship, while the main cause of their failure is dynamic overload. Damage to the horizontal joint is particularly critical, leading to the machine being halved in extreme cases. Joint failures have fatigue nature and begin with micro damages that propagate as a result of high-impact dynamic overloads. Ultimately, the micro damages turn into cracks in the structure, leading to permanent damage to the joint. Predictive detection of such malfunctions is the core of the research presented in this article.

The level of dynamic overloads that affect mining machines strictly depends on road conditions, machine load and the operator’s driving style. In practice, the roads can have different shapes, lengths, slopes and layouts of intersections. The special features of mining pavement are bumpiness, the presence of scattered rock blocks, mud and damaged surfaces. The technical condition of the road infrastructure is adversely affected not only by the number of machines operating in the mining area, but also by the operating and environmental conditions, as well as the quality of the blasting process in mining faces. The influence of the other factors remains unrecognized due to the lack of data enabling their identification in time and space. As mentioned before, the mediocre condition of roads generates dynamic overloads, the presence of which leads to the loss of stability of machine elements, especially structural nodes, which ultimately results in their damage. Tracking road conditions and dynamic overloads is very important for adjusting the proper design parameters to the specific conditions that occur in the underground mine, as well as for establishing the normative requirements for the self-propelled machines. Obtaining multi-dimensional insight requires the recognition of existing loads, the development of calculation procedures, conducting a series of experimental works, simulations, etc. In practice, self-propelled machines belong to a group of so-called heavy-duty machines. Their size and mass determine their dynamics and external load characteristics, including high-energy loads at low frequencies and low cycles [9]. In [10], the authors explained that the calculation instructions generally found in the literature do not take into account dynamic effects coefficients. The operating conditions in most cases introduce more energy into the structure than the specified design takes into account. This is the reason why it is necessary to identify the relationship between the dynamics of the supporting structure and the variability of the undercarriage loads, which gives direct information on the causes of component fatigue. In the case of mining machines, operational loads can very often have a percussive character [11,12,13]. As [6] states, dynamic shock loads have a decisive impact on the efficiency of a wheeled vehicle, internal devices and the psychophysical state of the vehicle driver.

Wheeled vehicles driving on roads, especially with high amplitudes of changes in the ground profile, are constantly subjected to dynamic effects. The nature of these loads has a complex structure. We can distinguish differences in value, duration, intensity or direction of action (vertically, transversely, longitudinally). Their main factors include:

- High and time-varying driving resistances resulting from road conditions and the observed force reactions;
- Operation of rotating elements (drive unit and chassis system);
- Actions of inertia forces (skidding, driving round bends, rapid acceleration, etc.);
- Dynamic loads occur as a result of moving at high speeds on various surfaces (especially on uneven surfaces) [9,14].

Moreover, the wheeled haulage process is not stationary. It is a cyclical process composed of several repetitive sub-processes: loading, driving with full cargo box, unloading and driving with empty cargo box. The operator can change the route, which is also associated with different road conditions. It is worth emphasizing that a lot of random events may occur during the operation, generating additional dynamic overloads (e.g., hitting the machine against the side of the excavation wall).

Research related to the factors and consequences of the impact of dynamic loads on vehicles has been carried out for many years. An interesting example is the vehicle scanning method (VSM) for bridges, which has been very popular recently. It is an indirect approach to measuring the frequency of bridges using sensors placed in vehicles. The most up-to-date overview of the results of the work carried out on this method is presented in [15]. Unfortunately, most of the other works mainly concern light vehicles and are oriented toward influencing drivers [16]. Very often, the conditions of the run tests differ from the actual conditions of machine operation. Most studies do not take into account the characteristics of changes in the profiles of various types of roads. The level of instantaneous dynamic loads occurring on the elements of the undercarriage of the machine resulting from the impact of the pavement is most often modeled with peak values of the amplitudes of their vertical acceleration. In practice, quantitative measures are also used to determine the vibration energy level of vehicle components, such as the effective value of changes in the vertical acceleration RMS. In [14], the authors presented research on the impact of road conditions on dynamic overloads in combat vehicles. In [17], the author provides an interesting tool for a comparative analysis of the level and frequency structure of machine component vibrations resulting from road conditions based on the power spectral density (PSD) of vertical accelerations. Similar research has been presented [18] on passenger-all-terrain vehicles. The tests were carried out on the basis of measuring the acceleration and deflection of the front axle suspension and the acceleration of the body frame for various road conditions.

However, the above methods allow only the monitoring of the level of dynamic overloads observed in various conditions. They do not provide information about the technical condition of structural nodes or the appearance of backlash. Our article focuses on the detection of damages caused by such deviations, which is based on the spectral entropy of vibration signals. In the case of the horizontal pivot of the self-propelled machine, backlash compensation can be reduced temporarily by tightening the nut connecting the front to the rear. In extreme cases, it is no longer possible to tighten the nut and its replacement is required. Early detection of damage allows for planning corrective work in advance and prevents failure related to joint breakage, which is crucial for safety and economic reasons.

The structure of the article is as follows: at the beginning, a description of the research is presented, indicating the need to detect damage to the joint of the haul truck, at the same time referring to the current research on similar problems. The research method with the use of the NGIMU sensor was presented. The analysis methodology based on the spectral entropy of vibrations is presented below. The method was applied to real data collected from several machines during experiments in the mine, showing that there are changes in the value of the spectral entropy difference related to the joint events.

## 2. Description of the Research

As mentioned earlier, the present research is focused on the method of early detection of joint damage in haul trucks. Detecting damage based on vibration analysis is a frequently researched topic, especially for rotating elements. However, there are few to none solutions dedicated strictly to self-propelled machines. Most of them concern engines or bearings and are focused on electromechanical devices or passenger vehicles. The problem with these machine parts seems to be simpler due to the possibility of determining the reference vibration frequency. For example, the article [19] uses the Short-Time Fourier Transform to analyze engine vibrations in order to detect failures caused by bearing currents. They showed the occurrence of a change in a specific frequency band depending on the increasing damage. In the article [20], the authors tested traditional methods for detecting and diagnosing ball bearing faults: FFT, Cepstrum, Amplitude Modulation, and Hilbert Transform. They indicated that, for the last two methods, the fault is slightly noticeable in the signal.

In the literature, there are many examples of the use of various types of signal entropy to detect damage to machines and structures. For example, in the article [21] a method of detection of looseness (backlash) of rail fasteners using amplitude entropy was proposed. The authors performed numerous experiments that caused mechanical vibration excitation. Using entropy, they showed that loosening caused slower vibration damping. The authors of the article [22] proposed a method for monitoring the technical condition of machines by measuring the approximate entropy for vibrations of an electric engine or bearing. They indicated that the number of frequency components increases with the deterioration of the technical condition of the machine, i.e., the entropy increases. There are also numerous examples of the use of spectral entropy to detect machine damage. In articles [23,24,25], spectral entropy was used to detect bearing failures. In previous works [26,27], spectral entropy was used to analyze damage of gears. In turn, the use of this method in examples of rotating machines can be read in [28,29]. In most of the examples cited, the methods of fault detection are based on determining the reference state of vibration for an operational machine and indicating changes after failure occurs. Additionally, the experiments were performed in controlled laboratory conditions, so the influence of random external factors was not taken into account.

**Figure 2:**
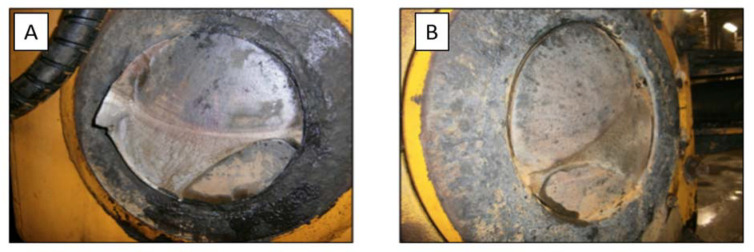
(**A**) Investigated technical object–haul truck, (**B**) typical operating conditions of an underground mine-the haulage route.

As mentioned above, joint damage usually begins with micro damage in the joint structure and can even end with the machine being halved. Photos that describe damage are presented in Figure 2. According to the designers of the researched machines, the main source of damage to the joints is loose nuts in the vicinity of its assembly points, which increases all forces acting on the joint. To counteract this phenomenon, they designed a new machine model with hydraulic nuts, but one such model with a damaged joint is already known.

**Figure 3:**
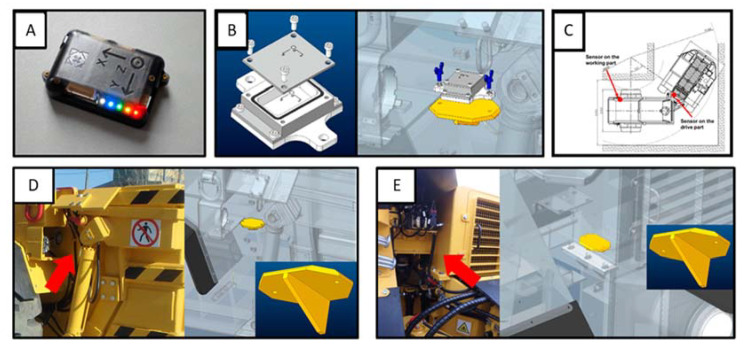
Data acquisition layer—main components: (**A**) NGIMU sensor unit; (**B**) Steel housing of the device and the method of installation on the machine; (**C**) Positions of the sensor marked with red dots; (**D**) Exact location (red arrow) of the sensor on the back of the machine; (**E**) Exact location (red arrow) of the sensor on the front of the machine.

In order to become more familiar with the joint damage, we proposed a measurement solution, which was later applied to some of the machines. Our method consists of using two IMU’s (Inertial Measurement Unit) from which one is mounted on the driving part and one on the working part (cargo box). In this solution, for both sensors, we used NGIMU (New Generation IMU) [30] along with custom-designed steel casing for their protection from the environment. The sensor itself, the steel protecting unit and the mounting points are presented in Figure 3. The entire research period lasted about 1 year, during which 16 machines from three different mines were examined, which allowed us to obtain almost 200 samples. During the examination period, each machine had both sensors installed on the first shift before leaving for work. The battery in the sensors, with the assumed measurement parameters, was sufficient to register about 2.5 working shifts (15 h). However, all measurements have been standardized to 2 working shifts, so a single sample is understood as the readings from two working shifts, both at the front and at the rear of the machine.

Each of the installed NGIMU sensors measured four quantities with a maximum frequency of 400 Hz:

- Acceleration–using the triaxial accelerometer with 16-bit resolution and 16 g range;
- Angular velocity–using the triaxial gyroscope with 16-bit resolution and 2000 °/s range;
- Magnetic field–using the triaxial magnetometer with 0.3 μT resolution and 1300 μT range. Unfortunately, the readings from the magnetometer were unusable due to the attenuation resulting from the housing;
- Humidity–using hygrometer with 0.008% resolution and 0–100% range.

Signals from the IMU can be quite heavily contaminated, e.g., due to missing measurements or outliers. In such cases, they can be repaired, for example, using Bayesian [31,32] methods. However, in our case, there was no such need. Due to the software, measurements from the NGIMU unit do not contain NaN values, probably because the sensor waits for the measurement to be fully completed. For this reason, problems may occur with the sampling rate instead. After analyzing all samples, we obtained an average sampling frequency of approximately 311.15 Hz and an average maximum time interval between two sensor readings in one session (8 h of machine work) of 0.1 s. As for the outliers, the signals were analyzed using the Z-score, which established the average percentage of outliers at a level of 0.18%. Taking into account the fact that the raw signal is segmented into fragments of several minutes (hundreds of thousands of measurements), these samples are collected together and processed later by statistics (spectral entropy) and that during these selected segments it is expected that the measured values are a stationary process, no need to implement additional preprocessing methods was found. 

After consultations with many engineers working in the construction and design of underground machines, it was determined that the fault of the joints was caused by dynamic overloads. These overloads increase significantly with the loosening of the joint assembly element (mostly the nuts). Therefore, the analyses carried out by us focused mainly on the signals most correlated with the overload phenomenon. Several factors influencing the forces to the greatest degree were also selected. These factors are:

- Machine working environment. It was established that in each mine and even in different mining departments (within one mine), different environmental conditions prevail. From the point of view of dynamic overloads, road maintenance conditions have a special impact. Research has shown that worse road conditions result in higher forces acting on joints and therefore at a larger risk of damage.
- Operator driving style. Each of the machine operators has a slightly different style of driving. People driving too dynamically cause significant vibrations, which are even more aggravated by the quality of the road.
- The type of operation performed. The work of the haul truck can be divided into work cycles, each of which has the following structure: driving with an empty box, loading the box, driving with a full box and unloading the box. Each of these operations has a different vibration characteristic. The biggest difference can be seen between driving with a full box and driving with an empty box, during which much greater vibrations are observed.
- Characteristics of the machine. After excluding all the above factors (assuming that the machines are moving in the same workplace, are driven by the same drivers and perform the same operation), it turns out that the machines still have different vibration characteristics. This means that the structural features of the machine, which are difficult to study, also have an influence.

**Figure 4:**
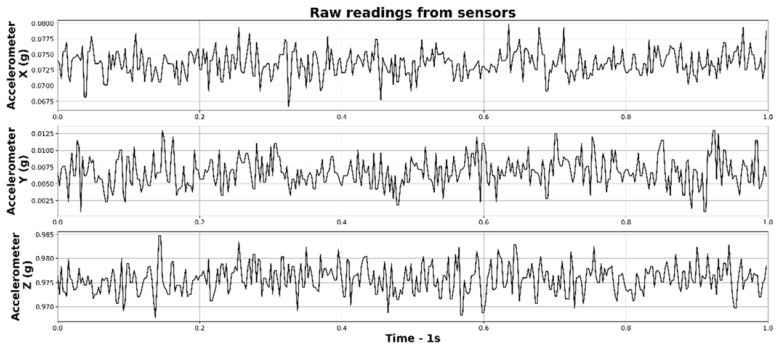
One second of accelerometer readings from the NGIMU unit.

As mentioned before, dynamic overloads (most probable cause of researched damage) are closely correlated to the vibrations affecting the machine. For this reason, only the accelerometer readings were taken into account. In standard situations, a uniaxial accelerometer is used to measure vibration (e.g., [22]). In this case, however, as the sources of vibration are dynamic and occur both outside and inside the machine, all 3 axes were used. One second sample of all accelerometer axes readings is presented in Figure 4.

## 3. Methodology

A significant number of different algorithms or estimators were applied to the data during the research, but most of them yielded little results. The results obtained by them were compared with the machine service books in order to find the relationship indicating damage. However, one measure showed convergence, and on its basis, the methodology described below was developed. This measure is the spectral entropy of the signal.

Spectral entropy is based on the information entropy (also known as Shannon Entropy) that was developed by mathematician Claude E. Shannon in 1948 [33]. Information entropy is the measure of information, choice and uncertainty and is a way of answering a question about the amount of information in a given message. In general, the Shannon entropy for the random variable x for n random events {x_1_,x_2_,x_3_,…,x_n_} can be written as follows (1).

(1) $$H=-K\sum_{i=1}^{n}p\left(x_{i}\right)\cdot \log_{2}p\left(x_{i}\right)$$

where p(x_i_) is probability of event x_i_, and $\sum_{i=1}^{n}p\left(x_{i}\right)=1$. K is a positive constant depending on the choice of measurement unit. Thus, the defined entropy has some properties. It reaches its maximum value when all events are equally probable (most unpredictable situation) and on the other hand, its minimum value (zero) is reached when the situation is certain (one event with probability of 1). Additionally, entropy increases as the events increases [23,33].

Shannon entropy has been successfully interpreted in a variety of ways and fields. In the case of signal processing, it can be used both in the time or frequency domain. In both cases, several methods for determining signal entropy have been developed. For example, Approximate Entropy (AnEn) [34] tries to determine the amount of regularity in time series using pattern-finding methods. A modification of this method is Sample Entropy (SampEn), which has the advantage of being independent of the sample length [35]. From all of these possible variants, spectral entropy was chosen with regard to the signal domain.

When considering vibration signals (accelerometer readings), spectral entropy can be used to study the signal in the context of occurring frequencies. Then, according to the Shannon entropy formula (1), the events x_i_ can be understood as individual frequencies, and the probability p(x_i_) as the frequency of their occurrence. The key task in spectral entropy is the determination of frequency distribution. One possible solution is to use the normalized Fourier transform, as was done by the authors of [23]. However, in the case of real, noisy signals in which there may be many overlapping frequencies, the authors decided that the better choice is to use power spectral density (PSD), as shown in [36].

The power spectral density can be estimated using a periodogram [37]. For the time series {X_t_}, where t ∈ {1,2,3,…,N} and frequency f the periodogram is defined by (2). Assuming that the PSD is to be used as a probability function, its normalization is required (3), which results in (4). Finally, the spectral entropy (SE) can be defined as (5), where $K=\frac{1}{\log_{2}n}$ so the SE variable is normalized to the interval (0, 1).


(2) $$P_{x}\left(f\right)=\frac{1}{N}\left[{\left(\sum_{t=1}^{N}X_{t}\cos\left(2\pi ft\right)\right)}^{2}+{\left(\sum_{t=1}^{N}X_{t}\sin\left(2\pi ft\right)\right)}^{2}\right]=\frac{1}{N}{\left|\sum_{t=1}^{N}X_{t}\exp\left(i2\pi ft\right)\right|}^{2}$$

(3) $$p\left(f_{i}\right)=\frac{P_{x}\left(f_{i}\right)}{\sum_{i=1}^{n}P_{X}\left(f_{i}\right)}$$

(4) $$\sum_{i=1}^{n}p\left(f_{i}\right)=1$$

(5) $$SE=-K\sum_{i=1}^{n}p\left(f_{i}\right)\cdot \log_{2}p\left(f_{i}\right)$$

Such defined spectral entropy will be 0 in case of pure sine wave and will gradually increase as the number of frequencies in the signal increases until it reaches 1 for white noise. Thus, the signal entropy describes the complexity and regularity of the signal and seems to be a good measure for the comparison of vibration signals.

**Figure 5:**
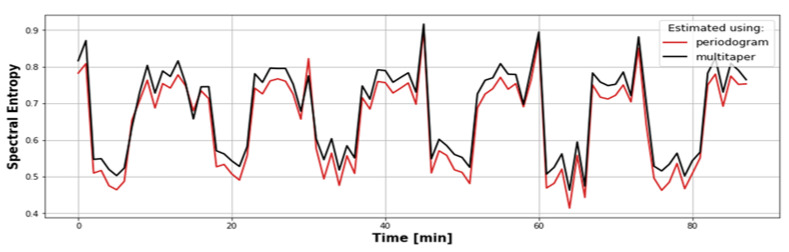
Comparison of spectral entropy estimated with the use of a periodogram and multitaper.

The use of periodorgam for PSD estimation is a popular method due to its simplicity. However, it is not without disadvantages; it is important to remember about the large variance of the periodogram. A way to deal with this problem is, for example, to use multitaper estimation [38]. It was decided to compare the spectral entropy determined using both methods. An exemplary result is shown in Figure 5, where the spectral entropy determined in the 1 min window for the fragment of the vibration signal recorded by the sensor placed on the machine is presented. It turned out that there are some value differences, but they are constant, which means that they will not affect the final results. Any fluctuations will also be leveled by averaging the values in the larger window. An additional advantage of the periodogram is the speed of the calculations. Multitaper requires several spectra determinations and averaging, which results in a longer computation time. For these reasons, it was decided to use an ordinary periodogram.

The aim of this work is to use the spectral entropy of haul truck vibrations to detect loosening or damage to the machine’s joint. Due to the large number of factors influencing the vibrations of a working machine, a direct comparison of the spectral entropy for an efficient and damaged machine would rather not be successful. These would be signals from two different shifts, where the machine was driven by different operators on different roads with different conditions. All these and other unknown factors affect the variability of the vibration signal. However, with two sensors mounted, one on the drive part of the machine (in front of the articulation) and the other on the working part (behind the articulation), it is possible to compare the vibrations from both sensors measured simultaneously. In other words, it is possible to analyze how vibrations are transmitted through a sensitive part of a machine. These signals, of course, will not be the same, but should be correlated with each other. The vibrations may be different, but if the state of the machine does not change, they should be transferred in the same way. Thus, the difference between the value of the spectral entropy of vibrations at the front and back of the machine should be constant. An increase or decrease in the entropy value for only one signal may indicate some irregularity. Being aware that the articulation is between the two vibration measurement points and that it is often damaged, the suggestion is that the articulation is related to irregularity. Thus, this method allows for the detection of damage to the machine articulation.

## 4. Application to Real Data

**Figure 6:**
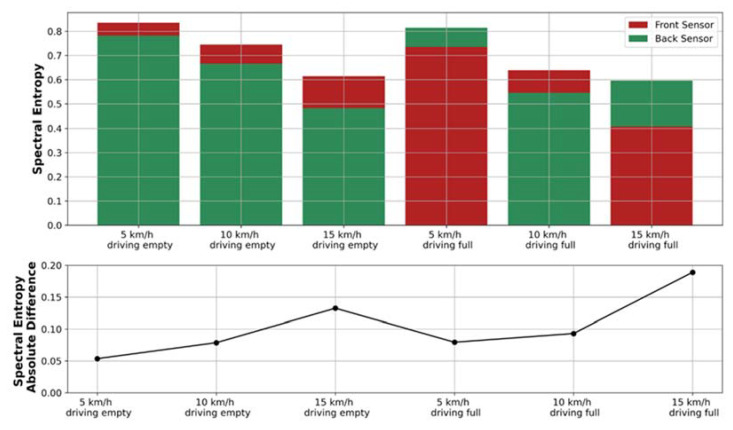
Entropy analysis during experimental driving over bumps. The spectral entropy of the Z-axis accelerometer for the front and back of the machine and the absolute difference between the entropy measured at the front and the back.

As mentioned earlier, the research presented in this article assumes the use of spectral entropy to analyze data from IMU sensors installed on haul trucks working in underground mines. This measure is highly dependent on several factors, the main ones being operator, speed, operation performed and the machine itself. These elements can create large differences between front and back readings, hence the need to take the difference between them. An example of these influencing factors is shown in Figure 6, where entropy was calculated with respect to machine speed and box status (empty/full). The upper graph shows a bar graph of the spectral entropy of vibrations measured at the front and back of the vehicle, depending on the factors while driving. It can be seen that the entropy values decrease with increasing velocity. The lower graph shows the absolute difference in entropy values between the front and rear of the vehicle. The entropy difference fluctuates, and its absolute value does not exceed 0.08. Thus, it can be concluded that, while the values of the spectral entropy may depend on various factors, the difference in spectral entropy between the front and back does not depend on such factors.

**Figure 7:**
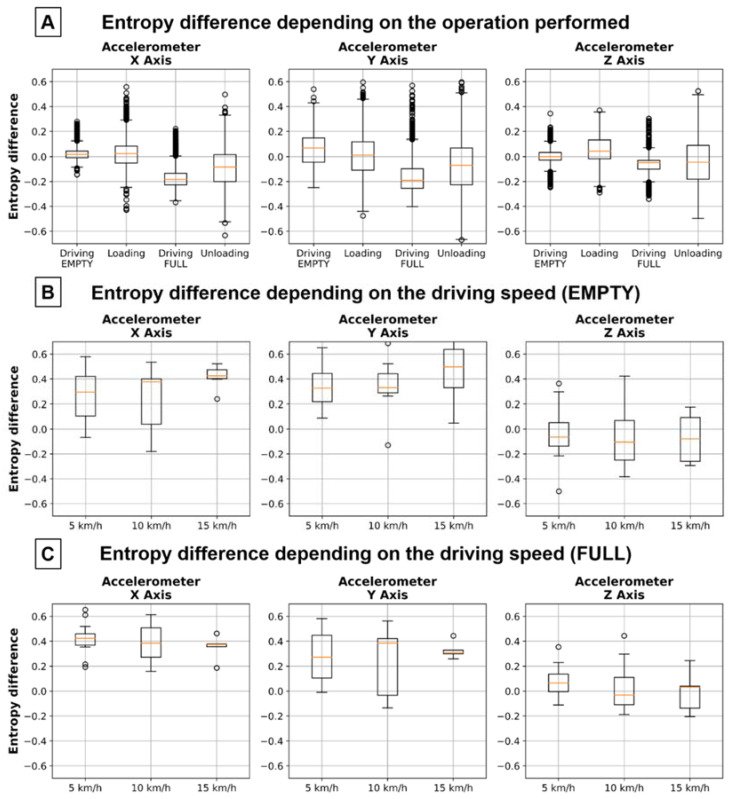
Comparison of the difference in entropy measured between the front and back, depending on the: (**A**)—operation performed, (**B**)—driving with different speeds with a full box, (**C**)—driving with different speeds with an empty box.

The difference of spectral entropy is shown in Figure 7 as boxplots with regard to operation performed and driving speed, divided into 3 accelerometer axes. Figure 7A shows the value of the entropy difference depending on the operation of the working cycles of a single machine. As the accelerometer in the Z axis should show the greatest correlation with the researched problem, the moments of driving with an empty/full box were selected as the best states for the analysis. When analyzing the other axes, it can be noticed that they have much larger possible positive values of the entropy difference (even over 0.6) than the Z axis (maximum 0.4). In addition, in most cases, there is a trend related to machine speed, which causes the values of the entropy difference to be more concentrated at the lowest and highest values.

Hundreds of samples and their entropies were analyzed during the entire period of the study, but in all of these, only a few samples had some form of event related to the failure under investigation. These events concerned a total of 6 machines: damage to the nut (1 sample), damage to the hydraulic nut (1 sample), tightening the nut (5 samples), and replacement of the nut (3 samples). In this case, machine 006/A was extremely informative, from which the most samples were collected. This machine, for yet unknown reason, has a greater tendency to break down than others, which allowed us to register as many as 5 events concerning joint damage and maintenance.

**Figure 8:**
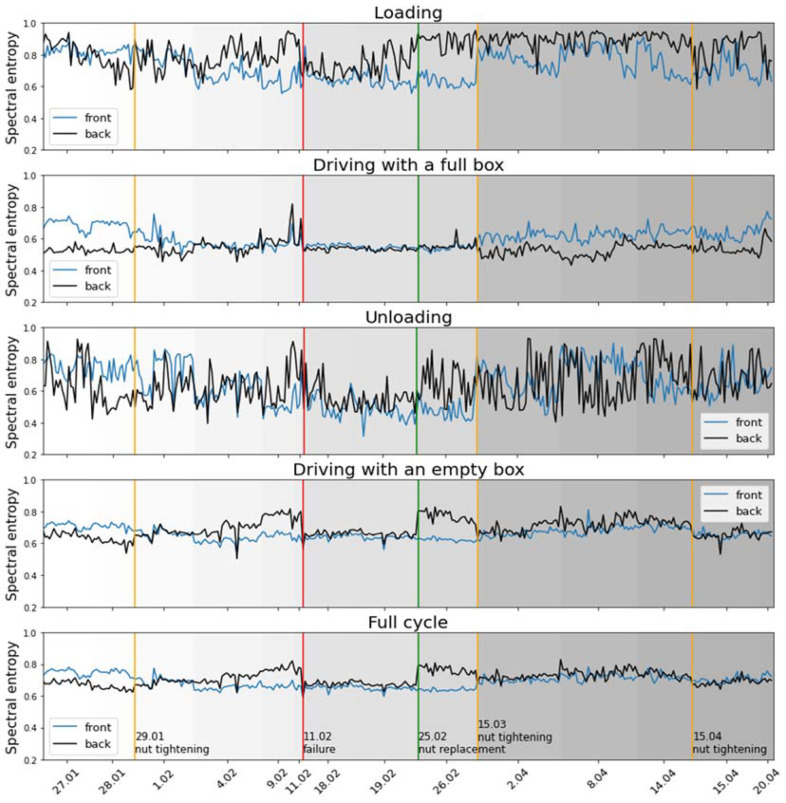
Spectral entropy of the Z axis accelerometer calculated once per cycle and a component operation for 14 selected days for machine 006/A.

When analyzing the entropy values of accelerometer Z from machine 006/A, some correlations to recorded events are visible. This is shown in Figure 8, where entropy values are computed over time with respect to the operation performed. The graphs show the value of entropy measured at the front and back of the vehicle, separately for the cases: loading, driving with a full box, unloading, driving with an empty box and the full cycle, i.e., without distinguishing between operational components. The event related to the maintenance of the joint has also been marked with vertical lines. It can be seen that for most events recorded, there is an increase in the difference between the back and front sensors in the days before such an event. This is especially seen in driving with an empty box operation. At critical moments, this difference is so large that it reaches 0.2, which is about 33% of the standard value. The value of the difference is usually higher before the event; however, one recorded replacement of the nut caused an increase in value that was later compensated for by tightening the nut. This behavior was not observed in healthy machines. The differences between back and front sensors did happen, but not on such a scale and they did not persist for longer times.

**Figure 9:**
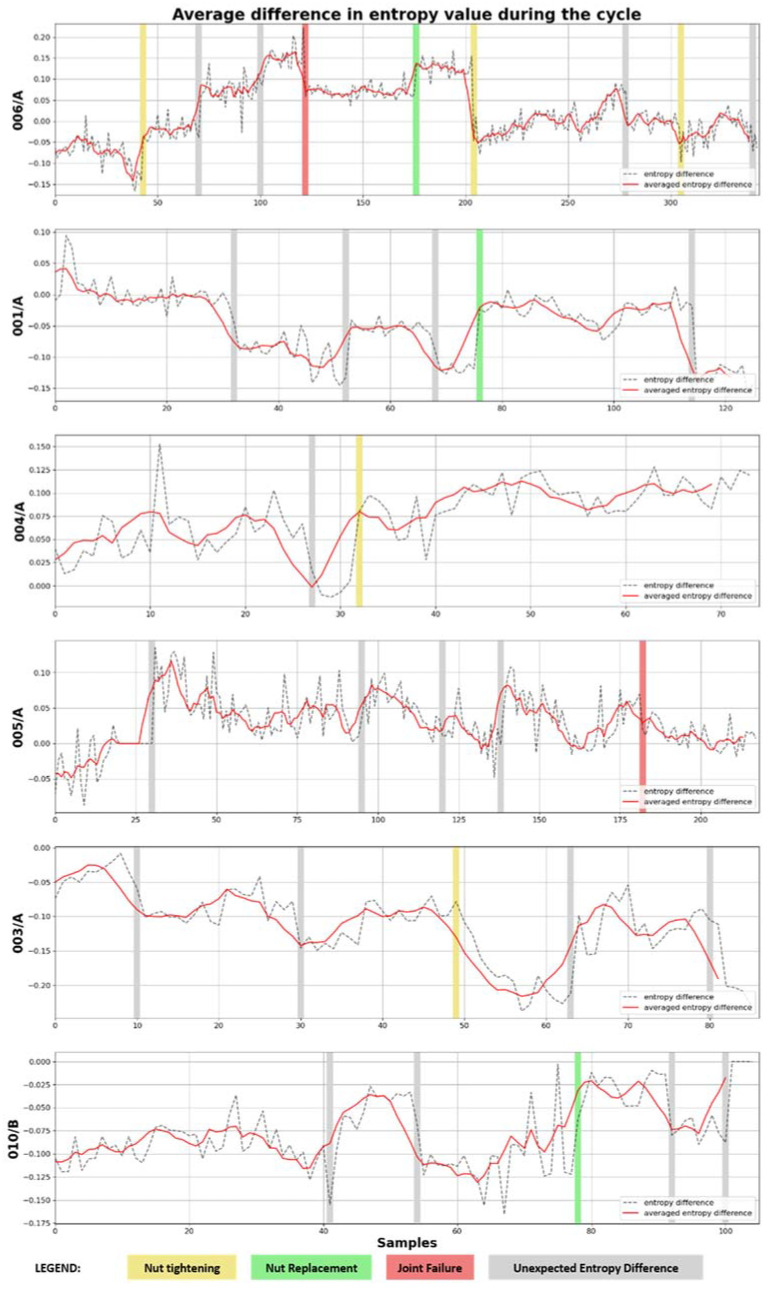
Comparison of the average difference in entropy values during the cycle for various machines.

For the purpose of further analysis of this behavior, it was decided to verify other failures in this regard. For these purposes, the average value of difference of spectral entropy between the back and front sensors was calculated for each cycle, but only from two components of the operation: driving with an empty and full cargo box. These differences are shown in Figure 9. Apart from the case of the 006/A machine discussed above, the charts also present other events related to the joint: the replacement of the nut in green, the tightening in yellow and the failure in red. At this point, the factor of errors in the maintenance and repair of the machine should also be taken into account. It is not a rule that tightening a nut will always improve its condition because it is a problematic activity (the location of the nut is difficult to access), and in some cases, the condition of the nuts may mean that tightening will bring expected results. Additionally, gray indicators have been added to the chart in places where no maintenance was recorded, but a large entropy difference change was detected.

When analyzing the graphs, it can be seen that practically every recorded event is reflected in a change in the level of the registered difference in entropy. The exception is the 005/A machine, which has a hydraulic nut, and the marked failure is a break in the nut’s operation. In addition, the signal waveform is completely different for this machine; it lacks the stabilization moments seen in other machines. In addition, one can also notice the fact that in the case of machines: 001/A, 003/A and 010/B, their entropy difference is definitely negative, which means that the values from the front sensor are higher than those from the rear sensor.

To provide an in-depth view of the problem, Table 1 presents the statistics of the difference in entropy for all machines. This statistic was computed for each machine by taking all the machine samples and then calculating the difference in entropy over the empty and full cargo box driving periods. All such periods were further combined into a single signal, as shown in Figure 9. On this signal, rapid changes in value were then detected by the step-detection algorithm, which was then categorized. The table therefore shows the jumps in the difference in entropy signals ∆x, broken down into increases and decreases, grouped by their values. In addition, the number of cycles (N), from which the values for the machine were calculated, were also given next to it. The jumps in statistics are related to the change in the way vibrations flow between the front and back of the vehicle, where larger ones may be caused by damage or backlash of the joint.

By analyzing the presented results, it can be concluded that all changes in the constant signal value by at least 0.05 should be the reason for further analyzes and investigation. At the same time, all deviations by smaller values are not likely to be related to a failure, provided that they do increase in time.

## 5. Conclusions

Not all damage to machines is immediately visible based on modern diagnostic methods. Often, damage begins with small structural changes that progress over time and will only be noticed when the machine is completely out of order. In such cases, symptoms very often appear just before failure. Very often it results from the lack of recognition of the genesis and evolution of the damage, selection of the measurement method and collection of the diagnostic base on the basis of which it is possible to propose effective reliability measures for inferring the technical condition. 

The article presents the problem of damage to the joint of the haul truck, which poses a serious threat to the health and life of employees and is also very expensive. Early failure detection avoids this risk. For this reason, a series of analyses was performed on the dynamic overloads occurring on the machines with the use of NGIMU inertial sensors located at the front and back of the vehicle. As it turned out, the problem is not easy because many different factors occurring while driving the machine in a mine have an influence on its dynamic overload. An analysis of the spectral entropy of vibrations (Z axis accelerometer) was performed, which showed that there was a correlation between the events related to the joint and the difference in entropy values measured at the front and back of the vehicle. Analyzing one joint fracture case, it was found that the entropy difference gradually increased before this event. Joint maintenance by tightening or replacing the nut has also been investigated, which has also shown that these activities typically reduce the spectral entropy difference. At the same time, it was shown that the difference in entropy values does not depend on factors such as the velocity or shape of the substrate.

## Figures and Tables

**Table 1:** Statistics of the difference in entropy for all machines.

Machine	Δx								
	Increases					Decreases			
	N	<0.05	>0.05	>0.10	>0.15	>−0.05	<−0.05	<−0.01	<−0.15
001/A	126	2	1	0	0	2	0	0	0
002/A	200	10	0	0	0	8	1	0	0
003/A	86	2	2	0	0	2	1	0	0
004/A	74	3	0	0	0	4	0	0	0
005/A	218	11	0	0	0	10	1	0	0
006/A	343	1	2	1	0	1	2	0	1
010/B	105	5	0	0	0	6	0	0	0
100/C	33	2	0	0	0	2	0	0	0
101/C	15	1	0	0	0	2	0	0	0
102/C	47	2	0	0	0	1	0	0	0
103/C	54	0	0	0	0	1	0	0	0
104/C	62	3	0	0	0	3	1	0	0
105/C	27	1	0	0	0	0	0	0	0
106/C	37	2	0	0	0	1	0	0	0
107/C	21	0	0	0	0	0	0	0	0

## Data Availability

Not Applicable.
